# Supplementary material for: Are carbon emissions trading and green financial instruments synergistic? -Comprehensive quantitative research based on content analysis
Source: PLoS One. 2024 Mar 7;19(3):e0298601. doi: 10.1371/journal.pone.0298601 (PMC10919601; doi:10.1371/journal.pone.0298601)
Supplement: S1 Data — (ZIP) [file pone.0298601.s001.zip › Data/地级市Z_F工作报告.zip!/╣π╢1⁄2/╜¡├┼╩╨/└┤╫╘╣1⁄2╓┌║┼í╛┬φ┐╦╩2╛▌═°í┐_2008.docx]

各位代表：

　　我代表市人民政府向大会作政府工作报告，请予审议，并请市政协各位委员和其他列席人员提出意见。

　　2007年工作回顾

　　2007年是本届政府的开局之年。我们面对在经济持续较快增长基础上如何实现又好又快发展的重大考验，面对在国家加大宏观调控力度下如何有效化解突出矛盾和问题的重大考验。我们坚持以科学发展观统领经济社会发展全局，克难奋进，开拓创新，较好地完成了市十三届人大一次会议确定的各项目标任务，实现了开好局、起好步的总体要求。

　　——综合实力跨上新台阶。

　　全市GDP突破1000亿元（1095.3亿元），增长15%，超过全省平均增速。人均GDP3641美元。三次产业比例演进为7.6：56：36.4。单位GDP能耗下降3%。地方财政一般预算收入62.4亿元，增长23.2%，占GDP的比重由上年的5.4%提高到5.7%。全社会固定资产投资324.5亿元，增长16.2%。进出口总额123亿美元，增长14%。社会消费品零售总额409.5亿元，增长16.2%。城镇居民人均可支配收入15149元，增长9.2%。农村居民人均纯收入5887元，增长5%。居民消费价格总水平上涨3.6%，低于全国、全省平均水平。

　　“四大百亿工程”开局良好。全市规模以上工业总产值2235.5亿元，增长24.7%；增加值520.6亿元，增长20.7%，增速超过全国、全省、珠三角平均水平。产值超30亿元的企业发展到8家，其中超100亿元企业1家，超50亿元企业2家；产值超50亿元的镇（街）11个，其中超100亿元的镇（街）5个；产值超100亿元的工业园区3个；产值超300亿元的产业集群3个。

　　新经济增长点加快形成。新增“中国船舶拆解基地”和“中国电能源产业基地”。汽车零部件和造纸产业集聚加快。船舶制造企业发展到14家，年生产能力超过30万载重吨。南洋船舶公司成为广东省最大的民营造船企业。台山核电站前期建设工作启动，川岛风电正式动工。中烟摩迪卷烟纸、富华重工、锦兴纺织、赫克力士化工等一批重点工业项目进展顺利。

　　自主创新能力进一步提高。全市新增高新技术企业25家，总数达190家。高新技术产品产值占工业总产值比重达30%，比上年提高2个百分点。启动省市联手共建化纤纺织产业升级工程。企业工程技术研发中心、专业镇分别新增22家和2个，总数分别发展到85家和18个。建立产学研结合研发平台或示范基地30多个，开展产学研结合项目300多项。申请专利超过3000件。新增2个中国名牌产品、1个中国驰名商标和5个中国免检产品。国家信息化示范城市建设取得新进展，信息化综合指数76.2%，比上年提高2.2个百分点。

　　内外源型经济协调发展。全市私营企业户数和注册资金分别达1.7万户、224.7亿元，分别增长9.6%和16.2%。规模以上民营工业企业完成工业增加值180亿元，增长22%，占全市工业的比重达37%。民资占固定资产投资比重50.1%，比上年提高6.1个百分点。对外贸易水平进一步提高，出口总额87亿美元，增长17.4%，获“中国摩托车出口基地”称号；进口总额36亿美元，增长6.4%。合同利用外资17.9亿美元，增长44.7%；实际吸收外商直接投资7.8亿美元，增长15.5%；新批超1000万美元项目71个，比上年增加23个。成功举办中国（江门）摩托车工业博览会暨经济技术贸易洽谈会、中国汽车零部件江门产业基地招商洽谈会等大型招商展会。

　　现代服务业发展水平显著提升。获中国最具投资价值新锐金融生态城市和福布斯中国大陆最佳商业城市称号。建立物流信息系统公共应用平台，14个物流项目建成投产。金融信贷增幅为5年来最好水平，证券交易额突破4000亿元。出台《关于推动市区企业改制上市的实施意见》，维达纸业在香港成功上市。成功举办中国（江门）侨乡旅游节，全年接待游客1460万人次，旅游总收入69.5亿元，分别增长38%和32%。完成房地产投资54.5亿元，增长88.3%，商品房销售面积和销售额分别增长22.9%和82%。

　　节能减排和节约集约用地初见成效。严把项目环保关，否批高污染项目96个。淘汰落后的立窑水泥生产能力80多万吨，关停小火电机组44.8万千瓦。完成工业污染源减排工程15项，全市新增生活污水处理能力9.3万吨/日。银洲湖纸业基地成为国家第二批循环经济试点园区。新会区成为广东省可持续发展实验区。节能技术推广力度加大。全市盘活闲置土地14572亩，工业项目土地投资强度比上年提高31%。

　　——城乡建设取得新进展。

　　滨江大道、江珠高速公路及双和公路建成通车，崖门5000吨级出海航道交工验收，珠三角城际轻轨、广珠铁路、江肇高速、江罗高速、中区大道等工程建设按计划推进，劳龙虎水道整治工程进展顺利。完成滨江新区规划和启动区城市设计，滨江新区开发建设迈出实质性步伐。以公交优先为重点的城市公共交通发展规划取得重要成果，新江门汽车总站建成启用，新会汽车客运站完成主体工程建设。生态环境保护成效明显，完成《江门生态市建设规划》和《江门市环境保护规划》。全市大气环境质量维持在国家二级标准，市区空气质量日报优良率96.7%；城市水域功能区和饮用水源水质达标率100%；建成区绿地率38.4%，绿化覆盖率41%，人均公园绿地面积9.5平方米。台山中华白海豚和恩平七星坑自然保护区升级为省级自然保护区。台山市成为省级林业生态县。

　　——

　　全市各级财政用于“三农”的投入为21.9亿元，增长26.9%。种粮补贴、能繁母猪补贴和购置农机补贴等一系列支农惠农政策全面落实。农业产业化步伐加快，产业化组织发展到277个，带动农户28万多户，占全市农户的39.8%，户均增收1380元。中低产田整治改造全面完成，水稻机耕率达92.5%。新发展无公害农产品生产基地35万亩，增长106%。村庄整治试点扎实推进示范村建设取得初步成效。镇级经济发展加快，镇级地方财政一般预算收入27.3亿元，增长27.5%，财政收入超千万元和超亿元的镇（街）分别达54个和5个。“异地发展”和经济发展“结对子”活动取得成效。年集体收入3万元以下的贫困村全部脱贫。

　　——文化名市建设实现新突破。

　　开平与村落获联合国第31届世界遗产大会表决通过，成为广东省首个世界文化遗产、中国首个华侨文化世界遗产。全市新增7个省非物质文化遗产项目。五邑华侨华人博物馆成为省级馆。以宋元崖门海战古战场、棠下良溪“后珠玑巷”、歇马“举人村”等为代表的历史文化，浮石飘色、荷塘纱龙等为代表的民俗文化，梁启超故居、陈白沙祠等为代表的人文文化，新会蔡李佛拳、鹤山咏春拳为代表的武术文化等文化品牌进一步打响。开平赤坎镇进入中国历史文化名镇之列。群众性精神文明创建活动蓬勃开展，组织举办文化服务百镇千村行、侨乡书香节、激情广场大家唱等一系列活动，社区图书馆、文化信息资源共享工程、“农家书屋”工程建设扎实推进。

　　——人民群众得到新实惠。

　　民生投入大幅增加，各级财政对民生投入总额27.6亿元，增长28.6%。全市包括2300名渔民子弟在内的约32万名农村孩子享受到全免费义务教育。全面完成义务教育学校危房改造任务。212万人参加农村合作医疗，参合率达90%，人均筹资标准和住院封顶线有所提高。市区人均居住建筑面积8平方米以下的低收入家庭住房问题得到解决。认真组织实施市人大城乡饮水安全保障工作议案，饮用水源保护和整治河涌污染的力度加大。农村公路硬底化新增151公里，完成4080户农村贫困户危房改造和1207户华侨农场危房改造。城镇最低生活保障标准提高到260至290元，农村提高到160元。对城镇低保家庭发放临时生活补贴。社保覆盖面持续扩大，全市基本养老保险缴费人数50.1万人，增长7.8%；基本医疗、失业、工伤、生育保险参保人数分别为52.4万人、43.3万人、39.1万人、29.2万人，分别增长9.9%、9%、8.7%、6.6%。

　　就业再就业工作成效明显。形成了覆盖全市的社区劳动保障就业服务网络，新增就业岗位2.3万个，安置城镇就业再就业3.2万人，其中下岗失业人员再就业7397人。开展“充分就业社区”创建活动和“百企扶百村”工程，重点帮扶“零就业家庭”和农村富余劳动力实现就业，登记在册的1073户“零就业家庭”全部实现了1人以上就业，转移农村劳动力2.8万人。城镇居民登记失业率为2.5%。

　　“平安江门”建设取得新成绩。组织开展打黑除恶、打击“两抢一盗”和游戏机赌博等一系列专项行动，对重要路段和部位实行电子监控，刑事发案率下降6.8%。全市43个社区通过省第一批“六好”平安和谐社区考核验收。蓬江区成为“全国和谐邻里建设示范城区”。落实领导包案制度，进一步健全预防和化解矛盾机制，信访总量、集体访、越级访继续下降。严格落实安全生产责任制和安全生产许可制度，加强对安全隐患的排查整治，完善应急预案体系和应急平台建设，安全生产形势平稳。妥善处置“6·15”九江大桥船撞桥梁事故。实现产品质量和食品安全专项整治目标。

　　——各项社会事业全面进步。

　　教育强市建设全面推进，新创建教育强镇（街）11个、国家级示范性普通高中2所、省一级普通高中2所，义务教育巩固提高，高中阶段教育普及加快，职业教育规模进一步扩大，五邑大学和江门职业技术学院有新发展。通过“国家卫生城市”复核，蓬江区荷塘镇成为全市首个“国家卫生镇”，中医药名市建设有新进展，农村和城市社区卫生服务得到加强，疾控中心建设上新台阶。成功举办市六运会和央视武林大会蔡李佛拳冠军争霸赛，群众性体育活动蓬勃开展。社会各界广泛参与慈善公益事业。全面完成省下达的人口计划。深入实施妇女和儿童发展纲要。人防、国防动员、民兵预备役和双拥工作进一步加强，实现“全国双拥模范城”四连冠。建立残疾人社区康复站（室）136个，7873名残疾人得到康复服务，蓬江区、江海区分别成为全国、全省残疾人社区康复示范区，城镇残疾人就业率89.3%。海外联谊、对外宣传、维护侨益与服务侨胞等工作有新进展。统计、新闻、出版、档案、方志、宗教、老龄、气象、红十字会等各项工作有新进步。

　　——政府自身建设进一步加强。

　　自觉接受市人大及其常委会和市政协的监督，坚持每季度市长约见人大代表制度，定期听取政协委员的意见建议，切实抓好人大代表建议和政协提案办理工作，共办结人大代表建议133件、政协提案124件,满意基本满意率分别达99.2%和100%。市直机关开展“三服务两促进”作风建设活动取得明显成效；全面完成ISO质量管理体系导入工作，科学管理水平进一步提高。“12345”政府服务热线覆盖所有市（区），“12345，有事找政府”成为各级政府为群众服务的品牌。向社会公布市县两级政府及职能部门领导手机号码，办好民生热线，为民服务渠道进一步拓宽。进一步减少和下放行政审批事项，健全重大事项社会公示制度和行政听证制度，依法行政和政务公开水平进一步提高，政务公开信息100%可在网上查询。实施推诿、搁置追究制，向社会公布8个行政过错典型案件。政府效能建设得到加强，市行政总汇受理业务近25万件，服务满意度逾98%。狠抓政府廉政建设和反腐败工作，加强效能监察和执法监察，市直部门行政审批环节实行电子视频监察，查处了一批违法违纪案件。

　　各位代表，过去一年我市经济社会的新发展，是在省委、省政府和市委的正确领导下，全市人民团结奋斗的结果；是各级人大及其常委会和政协监督支持的结果；是中央、省驻我市单位及军警部队、各民主党派、人民团体、社会各界人士、外来投资者和建设者、港澳台同胞、海外侨胞关心支持的结果。在此，我代表市人民政府，向关心支持参与江门经济社会发展建设的市民们、朋友们、侨胞们表示崇高的敬意和衷心的感谢！

　　我们也清醒地看到，市政府的工作与全市人民的期待还有不少差距，主要问题是：经济发展好中求快的步子迈得不够大，粗放型发展方式还未根本转变，产业竞争力不够强，现代服务业发展偏慢；区域发展不平衡问题依然突出，城乡居民收入增长不快、差距扩大；一些地方发生违法违规用地现象，征地拆迁、山林纠纷等问题引发的社会矛盾增加；教育、卫生、城市管理、社会治安等方面还有不少薄弱环节；政府服务满意度仍需进一步提高。我们必须高度重视这些问题，采取更有力的措施加以解决。

　　2008年工作任务

　　2008年是我市全面贯彻落实党的十七大精神，推动科学发展，促进社会和谐的重要一年。纵观国际国内形势，总体上有利于我市加快发展。当前，我市站在发展的新起点上，迎来新的发展机遇，同时也面临许多有待破解的发展难题。我们必须以新的思想大解放推动新的大发展。只有进一步解放思想，才能坚定破解发展难题的信心和勇气，才能找到破解发展难题的出路和办法，才能实现新跨越、赢在新起点。

　　今年市政府工作的指导思想是：高举中国特色社会主义伟大旗帜，以党的十七大、省委十届二次全会、市委十一届三次全会精神为指导，深入贯彻落实科学发展观，坚持“以好为先，好中求快”，进一步解放思想，深化改革，扩大开放，着力抓好“四大百亿工程”和重大项目建设，进一步转变发展方式，调整优化经济结构；着力改善民生和提高城乡居民收入水平，进一步促进社会和谐；着力转变作风和提高效能，进一步提高政府执行力，全面加快生态型现代化城市建设步伐，推动经济社会又好又快发展。

　　今年的工作，要紧紧围绕发展与和谐两大主题，实现以下预期目标：GDP增长13%，单位GDP能耗下降3.5%，二氧化硫和化学需氧量分别削减4%和3.5%，规模以上工业增加值增长16%，地方财政一般预算收入增长13.5%，全社会固定资产投资增长15%，社会消费品零售总额增长14%，外贸进出口总额增长10%，外商直接投资增长10%，城镇居民人均可支配收入增长9%，农村居民人均纯收入增长7%，城镇登记失业率控制在3.3%以内，人口自然增长率控制在7％。以内。

　　为实现上述目标，今年政府工作要重点抓好以下几个方面：

　　一、推进“四大百亿工程”，实现经济发展速度质量效益相协调。

　　以“四大百亿工程”为载体，推动经济又好又快发展，在不断优化产业结构的同时，做大经济总量，力争今年新增2家年产值超30亿元企业、3个产值超100亿元镇（街）、1个产值超100亿元园区、1个产值超300亿元产业。

　　提高招商选资水平，促进产业集聚和结构优化升级。突出抓好产业招商、大项目招商。进一步完善招商工作机制和招商信息共享平台，办好摩托车工业博览会，提高招商服务水平。重点抓好汽车零部件、造船及配件、电子信息、摩托车、纸业深加工、石化中下游产业和现代服务业招商。全力配合台山核电、台山电厂二期、新会电厂等投资超百亿元项目建设，确保28个超亿元的引资重点项目年内建成投产。打破地域界限，加大协调力度，落实产业发展规划，促进专业市场和公共技术创新服务平台资源共享，加快形成“数镇一带”产业发展新格局。坚持市场主导与政府引导相结合，建立税收分成和绩效考核等利益调节机制，鼓励各地按产业定位，引导项目定向投资落户，促进专业镇错位集聚发展，做大规模，做强特色，协同并进。年内增加财政收入超千万元镇(街)3个以上、超亿元镇(街)2个。

　　壮大民营经济规模，提高开放型经济发展水平。培育全民创业氛围，优化民营经济发展环境，各级政府当好民营经济发展的“引航员”、“护卫员”和“服务员”。

　　实施民营企业家素质提高工程和中小企业成长工程，鼓励民营企业走“以民引外”、“科技强企”和“上市发展”的路子，放开民营资本进入公用事业、基础设施和金融服务等领域。深化国有企业改革，强化国有资产监管，积极稳妥推进国有企业重组。提高对外经济贸易发展水平，优化出口结构，促进加工贸易转型升级，推动加工贸易企业扩大内销。鼓励企业走出去开展国际化经营。

　　加快现代服务业发展，积极扩大内需。充分发挥开平与村落世界文化遗产的品牌效应，加大旅游资源的宣传、整合力度，做好旅游大项目引进工作，推动旅游业大发展，力争今年全市旅游总收入超90亿元。城市中心区要实施“优二进三”战略，发展总部经济。加快新会银洲湖、台山广海湾、鹤山桃源和南新区等现代物流基地建设，推进江沙路汽车、新会不锈钢和开平水暖卫浴等专业市场建设。进一步发挥金融产业在国民经济中的作用，加强政银企合作，实施重点项目中长期合作计划，提高银行为中小企业服务水平，加快企业征信系统建设，发展证券期货保险业务。扶持优秀中小企业上市，培育3家以上企业准备上市。完成城市信用社退出市场工作，深化农村信用社改革，抓好恩平市村镇银行试点。继续积极引入外地金融机构，做好交通银行落户服务工作，营造良好的金融发展环境。拓展现代信息服务业和创意产业。调整住房供应结构，增加住房有效供给，促进房地产业健康发展。积极培育文化、休闲、健身等消费新热点，促进城乡居民消费。

　　二、推进自主创新，增强产业竞争力。

　　坚持以大中型企业为骨干，以中小企业为重点，加快自主创新体系建设，引导和支持创新要素向企业集聚，促进创新成果向现实生产力转化。

　　深化产学研合作。加大重点公共技术和关键技术攻关力度，把80%以上的科技专项经费用于组织实施重点工业项目。推动建立民间科技风险投资基金。鼓励企业参与省部产学研战略联盟。争取新建20家产学研联合基地和15家省市级工程中心。重点扶持50家大中型企业，培育25家以上高新技术企业，推动30家以上传统企业向科技型企业转型。组织实施省市级科技计划240项，全市专利申请量超3000件。

　　完善创新平台。推广开平水口水暖卫浴技术创新中心经验，加快摩托车、麦克风、不锈钢、五金卫浴、纺织服装等产业服务平台建设。建成国家摩托车及配件质量监督检验中心，推进广东华南精细化工研究院和广东广天机电工业研究院建设，力争全市企业工程技术研发中心突破100家。发挥好市科技创业中心二期作用，完成蓬江区科技孵化器建设。加强创新人才队伍建设，着力培养、引进优秀科技人才和团队。

　　继续实施名牌带动战略。加大名牌产品培育力度，引导名牌产品做强做大。加强知识产权和技术标准工作，鼓励优秀企业参与国家、国际标准制定，提升产品核心竞争力。力争新创中国名牌产品或驰名商标2个、省名牌产品3个、省著名商标5个。

　　三、推进建设，促进农民增收。

　　要把提高农民收入作为“三农”工作的重中之重，落实好各项支农惠农措施。

　　——投入重农。今年全市各级财政预算“三农”投入26.9亿元，增长22.9%。进一步加大农田基本建设的力度，完成5.27万亩国家农业综合开发土地治理项目和1.2万亩省人大关于加强农田水利基本建设议案整治农田目标，推进江新联围新会挡潮段等城乡水利防灾减灾和省人大水利议案工程建设，提高防灾抗灾能力。加大对“五改”工程、农村生活垃圾无害化处理和沼气应用的投入，全面完成农村公路硬底化建设。

　　——政策惠农。推进“异地发展”模式和经济发展“结对子”活动，扶持低收入村兴建集体物业，巩固贫困村脱贫成果，加快薄弱镇村经济发展。积极推进农村土地承包经营权流转，促进适度规模经营。加快完善各镇总体规划和控制性详细规划，逐步推进城乡基础设施建设一体化。健全基层民主管理机制，确保村（居）换届工作顺利完成。

　　——产业富农。合理布局农业产业，制定并实施农业产业总体规划、区域规划和专项规划。抓好现代农业综合示范基地建设，推进农业产业化经营，年内发展3家市级以上农业龙头企业和15家以上农民专业经济合作组织。大力发展畜牧养殖业和海洋产业，力争全市海洋产业总产值达到140亿元。加快现代林业发展，完成营造林21万亩，确保243万亩生态公益林不减少。巩固“万村千乡”市场工程，完善农村商品流通网络。

　　——科技强农。深入实施农村科技扶持计划和“信息直通车”工程。大力推广农业新品种、新技术，提高农业生产机械化水平和农产品良种覆盖率，新增无公害农产品生产基地15万亩。

　　——就业扶农。实施“千企扶千村”工程，加快农村劳动力向二、三产业转移，培训农村青年和农村富余劳动力4万人，培训后就业率达70%以上。

　　——保障稳农。逐步实施社会保障城乡一体化，落实被征地农民基本养老保险制度；继续提高农村最低生活保障标准；提高农村合作医疗保障水平，巩固提高全市参合率，住院报销补偿年封顶线不低于1.5万元；提高农村计划生育“即扎即奖”的奖励标准。解决农村饮水安全问题。加快华侨农场危房改造。

　　四、推进基础设施建设，打造现代化城市。

　　抓住我市被列入国家公路运输枢纽城市的机遇，做好交通综合运输枢纽规划的修编工作。抓好环城快速干道、东华大桥、礼东大桥、西环路隧道、东海路至南环路的规划建设，推进江新、新开高速公路和江顺大桥前期工作，配合推动珠三角城际轻轨、广珠铁路、江肇高速、江罗高速施工建设和佛开高速扩建等工程。加大国、省、县道改造力度，完成开阳高速公路恩城出口连接线工程和开平大道等项目，推进新中一级公路及新会龙马、连腰和大洞大桥等项目建设。抓好港口和航道建设，完成《江门港总体规划》修编和《江门港总体规划环境影响评价》编制工作，争取劳龙虎水道整治工程竣工，推进台山鱼塘港5万吨级码头和新会港二期工程2个万吨泊位及一批临港工业码头建设，启动崖门水道5000吨级航道整治工程，加大广海湾深水港招商建设的力度。按照《江门市滨江新区规划》与《滨江新区启动区城市设计》，推进滨江新区基础设施建设。加快南新区和银洲湖区域建设。实施中心城区公共交通发展规划，抓好市政道路建设，增加公交线路和班次，缓解中心城区交通拥挤和停车场不足状况。

　　五、推进节能减排，加强生态环境保护。

　　提高节约发展水平。实施《江门市可持续发展战略纲要》。深入开展节能减排全民行动，落实节能减排工作责任制，制订落实节能减排统计、监测和考核办法。加强对62家列入省“双千节能行动”重点耗能企业，以及2006年综合能耗超过1万吨标准煤企业节能工作的指导和监管。加快淘汰落后生产能力，按规定完成小火电、小钢铁、小造纸、小水泥等关停任务。加快省级循环经济试点城市和“推广新型清洁燃料试点城市”建设，支持新会区创建全国可持续发展实验区，推进清洁生产和再生资源回收利用体系建设，大力推广建筑节能和普及太阳能，全市新型墙体材料使用率达到70%以上。完成土地利用总体规划修编，严格执行土地利用年度计划，严把工业项目投资强度和容积率准入关，加快盘活闲置土地，推进旧村、旧区、旧厂整理，鼓励建设多层厂房，开展第二次土地调查。做好地质灾害防治工作。

　　提高环境保护水平。做好迎接“国家环保模范城市”和“国家园林城市”复查工作。全面实施环境保护规划、建设项目环保准入和主要污染物排放总量控制制度。在抓好各市(区)污水处理厂建设的同时，重点抓好镇级污水处理厂建设。加快市区第三生活垃圾处理场建设。加大机动车尾气污染整治力度。以铁的手腕治理工业废水偷排行为，坚持工业企业环境违法行为举报有奖制度，充分发挥新闻舆论的监督作用。做好第一次全国污染源普查工作。加快污染减排监控体系建设，确保6月底前完成23家国控污染源的在线监控。取缔一、二级饮用水源保护区内所有污水排放口，建立健全饮水安全保障应急机制，确保饮用水源水质。深入开展环境风险排查，增强突发环境事件应急处置能力。

　　提高生态建设水平。全面实施生态市建设规划，严格实施生态功能分区控制。继续抓好环境优美乡镇、生态示范村、绿色社区、环境友好企业等创建工作，创建市级以上卫生镇2个、卫生村40条。加快潭江及西江流域水源涵养林、自然保护区及野生动植物、湿地红树林及沿海防护林、绿色通道及农田林网、森林公园及城市林业等工程建设，加强森林资源、海洋生态环境与资源的管理和保护。

　　六、推进文化名市建设，发展繁荣侨乡文化。

　　加大文化名市建设力度，激发人民群众文化创造活力，增强侨乡文化软实力，兴起文化建设新高潮。

　　弘扬侨乡精神。坚持把社会主义核心价值体系融入侨乡精神文明建设全过程，深入开展群众性精神文明创建活动，力争成为“全国创建文明城市工作先进城市”。弘扬“开拓创新、文明开放、勤劳创业、团结奉献”的侨乡精神，特别是要形成浓厚的侨乡创业文化氛围。完善社会志愿服务体系，注重人文关怀，形成男女平等、尊老爱幼、互爱互助、见义勇为的社会风尚。

　　激发侨乡文化活力。加强侨乡文化的研究与宣传，营造有利于出精品、出人才、出效益的环境。推进开平与村落的保护开发，办好华人嘉年华活动和首届世界江门五邑青年大会，进一步打响具有五邑特色的华侨文化、历史文化、名人文化、民俗文化、武术文化等文化品牌。做好文化典籍整理和非物质文化遗产、文物普查工作，加强历史文化遗产保护。继续做好江门长堤历史街区、江门新宁铁路遗址、台山洋楼、开平赤坎古镇等华侨建筑群，以及良溪“后珠玑巷”、歇马“举人村”等历史文化的调查、挖掘、整理和申报升级。积极发展新闻出版、广播影视、文学艺术事业，壮大印刷、艺术培训、文化娱乐等文化产业，加快广东省影视动漫产业园建设和影视动漫产业发展。加强文化市场建设和管理，营造健康有序的文化市场环境。

　　丰富群众文化生活。弘扬中华优秀传统文化，创作和推出更多具有鲜明侨乡特色、反映时代特征、群众喜闻乐见的文化精品。加大公益性文化事业投入，加强文化馆、图书馆、博物馆和社区文化站建设，建成江门广播电视中心、市新青少年宫一期，办好五邑华侨华人博物馆和江门美术馆馆展。继续推进文化体育设施共享工程。构建公共文化服务体系，广泛开展文化下乡、文化进社区活动，推进“农家书屋”工程、文化信息资源共享工程，丰富农村和偏远地区及进城务工人员的精神文化生活。

　　七、推进和谐江门建设，着力改善民生。

　　坚持把民生问题作为政府工作首要解决的问题，确保当年新增财力的60%以上用于发展社会事业和改善民生。扩大公共服务，完善社会管理，促进社会公平正义，努力使全体市民学有所教、劳有所得、病有所医、老有所养、住有所居。今年各级财政对改善民生的投入总额33.4亿元，增长21%。

　　办好人民满意的教育。全市各级财政预算教育投入16.5亿元，增长18.2%。加快教育强市建设，创建3个教育强区、16个教育强镇(街)。提高五邑大学办学水平，做好五邑大学本科教学评估工作，继续办好江门职业技术学院和江门电视大学。加快高中阶段教育资源布局调整。大力发展职业技术教育。全面实施城乡免费义务教育。进一步理顺市区义务教育阶段办学体制。重视学前教育，发展特殊教育。

　　千方百计促进就业。完善扶持创业的政策和就业服务体系，安置城镇劳动力3万人。认真贯彻实施《劳动合同法》和《就业促进法》，提高最低工资标准，推行工资集体协商，促进企业建立合理的工资增长机制。深化“充分就业社区”创建活动，完善就业援助制度，加强就业技能培训，重点帮助下岗人员、低保家庭、残疾人和退役士兵就业，援助就业困难人员4000人。抓好高技能人才队伍建设，培养高级工1500人、技师以上500人。做好高校毕业生就业工作。

　　建立全覆盖的社会保障体系。全市各级财政预算社会保障投入4.4亿元，增长31%。进一步扩大社会保险覆盖面，今年6月底前全市参加养老保险的实际人数比上年增加4%，进一步做实个人帐户。建立城镇居民基本医疗保险制度，重点解决好60岁以上、18岁以下的城镇居民医疗保险问题。提高最低生活保障标准。构建住房保障体系，加快廉租房、经济适用房建设，并结合补贴房租的办法，解决低收入家庭住房困难。加强老龄工作，办好镇(街)敬老院。做好优抚安置工作。大力发展社会福利和慈善事业。加强价格监管，规范价格行为，保障市场供应。

　　加快医药卫生事业发展。全市各级财政预算公共卫生投入3.6亿元，增长25.8%。以医疗卫生服务、医疗保障、药品供应保障体制改革为重点，推进城乡医疗卫生服务体系建设。开展社区卫生服务试点和镇卫生院改革试点，健全农村三级卫生服务网络。完善疾病防控体系。建立健全医疗救助制度。加强卫生监督执法和药品监管体系建设。深入开展爱国卫生运动。发展妇幼卫生事业。推进中医药名市建设。

　　完善社会管理。积极稳妥推进城市管理综合行政执法改革，推广“数字城管”，探索城市“无缝化”和长效化管理模式。落实维稳目标管理责任，妥善化解人民内部矛盾。坚持领导包案、信访联席会议制度，完善市直部门领导公开接访制度。健全调解、接访、听证、仲裁机制，引导群众按法律程序合理表达利益诉求。加强出租屋、流动人口的服务和管理。探索区域治安防控合作机制，加快社会治安视频监控系统建设，严厉打击黑恶势力、“两抢一盗”和制毒贩毒等违法犯罪活动。做好监狱劳教和社区矫正工作。加强平安和谐社区建设，力争全市“六好”平安和谐社区占60%以上。

　　强化安全生产和食品药品安全监管。加大安全生产投入。实施安全生产重点监控企业公示制度、安全生产隐患和违法行为举报有奖制度。全面推进企业安全标准化建设，完善重大危险源安全管理信息系统，加大重点领域、重点行业、重点部位排查整改力度，坚决遏制重特大安全事故发生。建立产品质量和食品安全监管长效机制。完善应急管理机制，提高防范和处置突发公共事件能力。

　　严格落实人口与计划生育目标管理责任制，完善层级动态管理机制，推广免费婚检，提高出生人口素质。切实保障妇女、未成年人合法权益。大力发展体育事业和体育产业，广泛开展全民健身活动，以实际行动迎接北京奥运会召开。落实双拥和民兵预备役工作。做好侨务、外事、港澳台事务、统计、人防、气象、宗教、档案、方志等工作。

　　八、推进思想解放，提高政府建设水平。

　　新形势新任务，对政府自身建设提出了新要求。我市要站在新的起点上实现新一轮大发展，政府自身建设必须上新水平。

　　思想解放上新水平。广泛开展“思想解放大讨论”活动，大力破除小进则满、因循守旧、怨天尤人、怕担风险等心态的束缚，牢固树立忧患意识、机遇意识、创新意识和进取意识，始终保持昂扬向上、奋发有为的精神状态。围绕新一轮发展加强学习，以学习适应形势；围绕新一轮发展加强调查研究，用改革的思路和办法解决发展中的问题；围绕新一轮发展创造性地开展工作，不断提高贯彻落实科学发展观的能力和水平。

　　科学决策上新水平。深入推进政务公开，政府决策坚持公众参与和专家论证相结合，认真听取人大代表、政协委员、各民主党派、人民团体、无党派人士的建议和意见，畅通人民群众发表意见、反映问题的渠道。制订2020年远景经济社会发展纲要。做好主体功能区规划编制”规划中期评估及“十二五”规划前期研究工作。坚持经济形势分析联席会议制度、经济社会发展顾问咨询制度和重大事项征询意见制度，完善行政决策纠错机制。

　　依法行政上新水平。深入贯彻行政许可法，深化行政审批制度改革，进一步清理行政审批许可事项，简化审批环节，加强审批许可监督。严格执行重大决策合法性论证及评估反馈机制，落实行政执法责任制，加强行政复议和行政应诉工作。完善人大议案、建议和政协提案办理工作机制，提高办理质量。大力发展电子政务，实现政府部门间信息互通和资源共享。精简和规范各类议事协调机构及其办事机构，减少行政层次，降低行政成本。加快推进事业单位分类改革。

　　为民服务上新水平。扩大公共服务，完善公共财政体系，建设服务型政府。提升ISO质量管理体系运行监管水平。完善市县两级政府及职能部门领导手机信息事项办理程序，健全“12345”政府服务热线办理机制，提高办理满意度。推进以公共服务为主要内容的政府绩效评估和行政问责制度，严格执行推诿、搁置等行政过错责任追究制和限时办结制。切实保障人民群众对政府工作的知情权、参与权、表达权和监督权。

　　作风建设上新水平。坚持新时期群众路线，强化群众观念，以群众满意作为政府作风建设的出发点和落脚点，开展“机关作风建设满意年”活动，将“满意度”评价与ISO质量管理体系运行情况、“12345”政府服务热线和信访办理情况、行政总汇服务事项办理情况挂钩，将机关作风的检查评价常态化。大力弘扬求真务实的工作作风，开短会，发短文，讲短话，遏制“文山会海”。加强公务员队伍建设，提高公务员综合素质，营造团结和谐的干事氛围。落实廉政责任制，规范各级领导干部从政行为。坚决整治损害群众利益的不正之风，依法治理商业贿赂。严格执行建设工程项目招投标、土地使用权出让、产权交易、政府采购等制度，从源头上防止腐败。

　　各位代表！坚持科学发展，促进社会和谐，让全市人民安居乐业幸福安康，是我们光荣而艰巨的历史使命。让我们在省委、省政府和市委的正确领导下，在市人大、市政协的监督支持下，高举中国特色社会主义伟大旗帜，深入贯彻落实科学发展观，紧紧依靠全市人民，解放思想，同心同德，开拓进取，励精图治，为在新的起点上实现新的大发展而努力奋斗！
